# Multi-Protein Profiling Reveals High Nuclear KFL-4 Expression as a Predictor of Poor Overall Survival in Breast Cancer: A Retrospective Cohort Study

**DOI:** 10.3390/ijms27062576

**Published:** 2026-03-11

**Authors:** Mariz Kasoha, Bashar Haj Hamoud, Rainer M. Bohle, Barbara Linxweiler, Raphaela Bosch, Gilbert Georg Klamminger, Gilda Schmidt, Erich-Franz Solomayer, Meletios P. Nigdelis

**Affiliations:** 1 Department of Gynecology, Obstetrics and Reproductive Medicine, Saarland University Medical Center, 66421 Homburg, Germany; 2 Institute of General and Special Pathology, University Medical School of Saarland, 66421 Homburg, Germany; 3 Department of Obstetrics and Gynecology, University Medical Center of the Johannes Gutenberg University Mainz, 55131 Mainz, Germany

**Keywords:** breast cancer, KLF-4, KLF-5, epithelial–mesenchymal transition, immunohistochemistry, tissue microarray, prognostic biomarkers

## Abstract

Following the establishment of the four molecular subtypes of breast cancer, additional biomarkers are required to further refine prognostication and patient stratification. Krüppel-like factors (KLFs), components of Wnt signaling, estrogen receptor beta (ERβ) isoforms, cyclin D1, and E-cadherin have been implicated in epithelial–mesenchymal transition, tumor proliferation, and disease progression. In this monocentric retrospective cohort study, tissue microarrays from 153 patients with histologically confirmed breast cancer were analyzed by immunohistochemistry to assess the expression of cytoplasmic Dkk1, β-catenin, and E-cadherin, as well as nuclear cyclin D1, KLF-4, KLF-5, and ERβ isoforms, using the Remmele and Stegner immunoreactive score. Associations between protein expression patterns with clinicopathological characteristics and survival outcomes using univariable and multivariable Cox regression analyses were examined. High cytoplasmic E-cadherin expression was associated with improved overall survival [hazard ratio (HR) 0.37, 95% confidence interval (95% CI) 0.18–0.77, *p* = 0.008], whereas high nuclear expression of KLF-4 (HR 2.63, 95% CI 1.32–5.22, *p* = 0.006) and KLF-5 (HR 2.16, 95% CI 1.01–4.65, *p* = 0.048) was associated with reduced overall survival. High ERβ1 expression showed a marginally protective association with the development of metastases (log-rank test *p* = 0.045). Importantly, nuclear KLF-4 expression remained independently associated with adverse overall survival after adjustment for tumor stage, lymph node status, molecular subtype, and other molecular markers (adjusted HR 4.09, 95% CI 1.93–8.67, *p* < 0.001). These findings identify nuclear KLF-4 as an adverse prognostic marker in breast cancer and support its potential relevance for molecular patient stratification.

## 1. Introduction

Carcinoma of the breast constitutes the most common malignancy among women accounting for the largest number of cancer-associated deaths in almost all parts of the world [1,2,3]. Based on the most recent analysis of the Global Cancer Observatory, more than 650,000 women have died of breast cancer (BC) in 2022, underscoring the need to bolster the status of diagnostics and therapeutics, leading to individualized management strategies both in early and advanced disease [2].

BC treatment is comprised primarily of three pillars, namely operative therapy (including surgery of the breast and the axilla), medical therapy (including endocrine, neoadjuvant, or adjuvant chemotherapy and targeted therapies), and radiation therapy [4,5,6]. Notably, targeted therapies constitute the most evolving field, which is fueled by molecular research. Significant targeted therapies that have already been introduced in clinical routines include monoclonal antibodies and antibody-drug conjugates against Her2/neu (e.g., trastuzumab, pertuzumab, trastuzumab-emtansin) [7]; immune-checkpoint inhibitors, for example, pembrolizumab targeting programmed death-1 (PD-1) receptors [8]; cyclin-dependent kinases 4 and 6 (CDK4/6) inhibitors, such as ribociclib or abemaciclib [9,10]; and poly (ADP-ribose) polymerase inhibitors (PARPi) for BC patients with germline BRCA1/2 mutations [11].

In essence, all the above-mentioned treatments are based on the four molecular subtypes of BC, luminal A and B, Her2-enriched, and triple negative BC (TNBC), which constitute the most significant step towards personalization [12,13]. Adding to this, genomic technologies have recently provided tools that allow for tailoring of treatments strategies. For example, OncotypeDX and EndoPredict provide a patient-specific score assessing the additional benefit of chemotherapy in specific patients with hormone receptor positive BC [14,15]. Moreover, additional methodologies—including tissue microarrays, liquid biopsies, and serum biomarker profiling—have been investigated to refine prognostication and therapeutic decision-making [16,17].

As far as molecular events are concerned, loss of cellular adhesion capacity leading to the hallmark of epithelial-mesenchymal transition (EMT) and sustained proliferation signaling are critical processes that lead to tumor progression and metastases [18]. Regarding the former, components of adherent junctions, including E-cadherin, a cell-cell adhesion transmembrane glycoprotein, and β-catenin, which links E-cadherin to the actin cytoskeleton and functions as a transcriptional coactivator in the WNT signaling pathway, influencing embryogenesis, stem cell regulation, and EMT, have been studied in the pathogenesis and prognosis of BC [19]. More specifically, recent proteomic evidence has demonstrated their negative prognostic role in ductal BC [20].

Dickkopf-1 (Dkk1), a member of the Dickkopf family, inhibits the canonical Wnt/β-catenin signaling pathway by binding to Frizzled receptors and low-density lipoprotein receptor-related proteins 5 and 6 (LRP5/6). It also functions in the β-catenin-independent Wnt pathway and the Dkk1/cytoskeleton-associated protein 4 (CKAP4) signaling axis, influencing embryonic development, osteogenesis, and organogenesis [21]. Our group has shown that Dkk1 contributes to the development of osseous metastasis and modulates chemotherapy response in TNBC [22,23,24].

Another molecule family that remains underrepresented in the pathophysiology of BC are the Krüppel-like factors (KLF), a family of at least 17 DNA binding transcriptional factors involved in cellular differentiation, apoptosis, proliferation, migration, inflammation, pluripotency, and cancer stem cell regulation [25]. This has been demonstrated for some of the members of this family, as previous investigations have demonstrated the possible association of nuclear KLF-4 with aggressive biology in early BC [26]. Finally, despite the predominance of estrogen receptor-alpha (ER-α) and progesterone receptor (PR) in the pathogenesis of luminal neoplasms, estrogen receptor-beta (ER-β) and estrogen-related receptors (ERRs) have been demonstrated to exert a tumor-suppressing effect in cellular proliferation and, surprisingly, have been implied in the pathogenesis and prognosis of TNBC [27,28].

Given this complex molecular interplay, exploring the expression of these proteins using tissue microarray analysis may provide valuable prognostic insights and contribute to the refinement of personalized treatment strategies for BC patients. The objective of this retrospective cohort study was to evaluate the association between the expression of a panel of proteins implicated in BC carcinogenesis and clinical oncologic outcomes of affected individuals.

## 2. Results

### 2.1. Demographic Characteristics

A total of 153 patients constituted the study population. Median patient age was 55.0 years, with an interquartile range of 47.0–64.0 years. Ninety-eight patients (64%) were postmenopausal. Regarding the molecular subtypes, 82 cases (53.6%) were TNBC, followed by luminal B neoplasms in 23.5% of the cases. At the time of diagnosis, 59 patients (38.8%) had T1 disease, while lymph node positive tumors were observed in 72 patients (48%). A total of 85 cases (55.6%) underwent neoadjuvant chemotherapy. Regarding regression scores, 27 patients (31.8%) had a score of 0 to 1, 24 patients (28.2%) had a score of 2–3, and 34 patients (40%) were assigned a score of 4. In terms of operative technique, 114 patients (74.5%) underwent breast-conserving therapy (Table 1). During follow-up [median (range): 80 (45–119) months], 36 (23.5%) patients developed distant metastases in other organs, while 34 (22.2%) patients died.

### 2.2. Expression Levels of Different Molecules in BC Tissues

Table S1 presents the expression levels (according to Remmele and Stegner) of different molecules in the studied BC tissues. Cytoplasmic Dkk1 expression levels were mostly moderate (68 cases, 44.4%) followed by weak ones (62 cases, 40.5%). Cytoplasmic β-catenin levels were mostly moderate or strong with 61 cases (39.9%), while most E-cadherin cases demonstrated moderate staining, with 75 cases (49%).

Regarding the molecules with nuclear expression, mostly negative staining patterns were observed for KLF-4 [72 patients (47.1%)], KLF-5 [99 patients (64.7%)], and cyclin D1 [98 patients (64.1%)]. On the contrary, ER-β1 and ER-β2 demonstrated primarily strong patterns, while for ER-β5, most cases, namely 88 cases (57.5%), demonstrated moderate staining.

### 2.3. Correlation Analyses Between Protein Expression Scores

Kendall’s Tau-b correlation coefficients along with corresponding *p*-values between the expression levels of each protein are depicted in Table S2. Apart from significant positive correlations between different isoforms of estrogen receptors, other significant positive correlations included cytoplasmic E-cadherin with cytoplasmic ß-catenin expression (*p* < 0.001), nuclear cyclin D1 with Dkk1 (*p* < 0.001), and cytoplasmic E-cadherin (*p* = 0.015). KLF-4 was positively associated with nuclear ER-β1 (*p* = 0.006) and ER-β2 expression (*p* < 0.001). KLF-5 expression was negatively associated with nuclear PR (*p* < 0.001) and nuclear ER-α (*p* < 0.001) and positively associated with nuclear ER-β5 (*p* = 0.014) expression.

### 2.4. Associations Between Tested Protein Expression Levels and Clinicopathological Parameters

**Figure 1:**
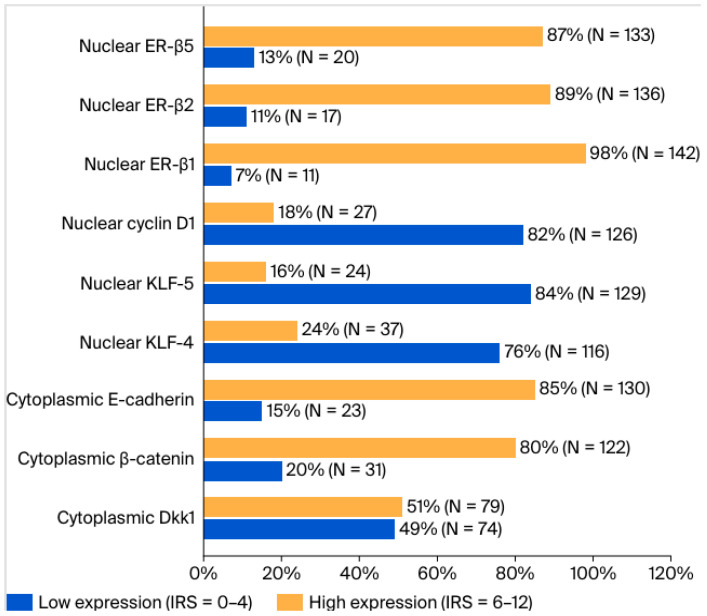
Expression levels of tested proteins dichotomized based on IRS. Cases with an IRS of 0–4 were classified as low expression, while those with an IRS of 6–12 were considered high expression. Data are presented as percentage and number of cases (N).

To assess the differences in clinicopathological parameters in different expression groups of each protein, cases were divided into two subgroups: one demonstrating low expression, corresponding to IRS scores 0–4, and the other with high expression levels, corresponding to IRS scores 6–12 (Figure 1). The results of these comparisons are depicted in Table S3. Among the studied cases, G3 tumors were more likely to demonstrate high nuclear KLF-5 expression compared to G1–2 tumors (24% versus 6%, respectively, *p* = 0.003). Compared with other molecular subtypes, TNBC cases were more likely to demonstrate low expression levels of cytoplasmic Dkk1 (*p* < 0.001), cytoplasmic E-cadherin (*p* = 0.003), nuclear ER-β1 (*p* = 0.011), and nuclear ER-β2 (*p* = 0.003). TNBC tumors were more likely to demonstrate high expression levels of nuclear KLF-5 compared to all other molecular types (*p* < 0.001). Moreover, tumors with a Ki67-index >14% were more likely to have high expression levels of KLF-5 (*p* = 0.014) and low expression levels of ER-β2 (*p* = 0.042) compared with those with Ki67-index ≤ 14%.

### 2.5. Survival Analyses

**Figure 2:**
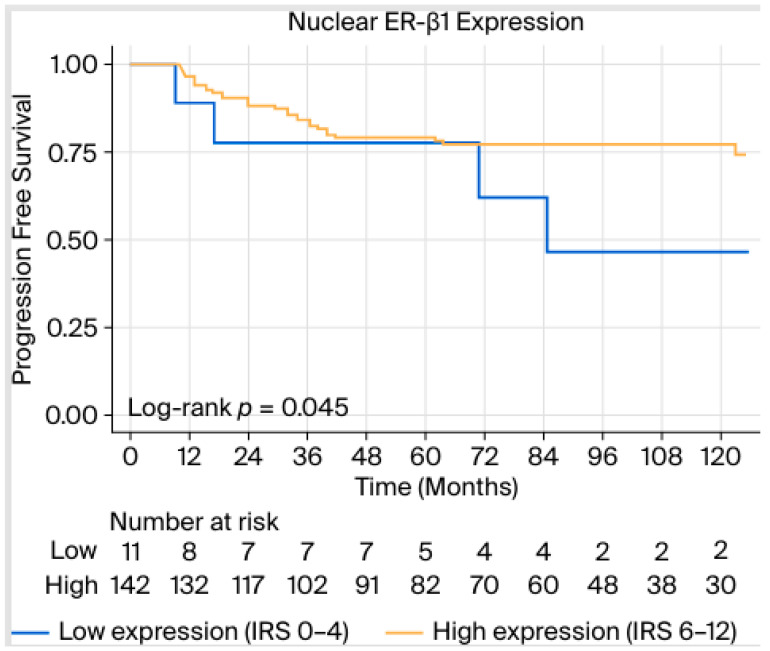
Kaplan–Meier curve and *p*-value corresponding to log-rank test between progression-free survival (PFS) and nuclear ER-β1 expression.

Table 2 and Table 3 demonstrate the results of the univariable and multivariable cox-proportional hazards models for PFS (i.e., development of metastases and/or tumor recurrence) and OS. We demonstrated significant associations of PFS with T2–4 compared with T1 tumors [hazard ratio (HR) 2.30, 95% confidence interval (CI) 1.08–4.90, *p* = 0.031], positive lymph node involvement (HR 4.27, 95% CI 1.97–9.28, *p* < 0.001), and TNBC compared with all other molecular subtypes (HR 3.60, 95% CI 1.63–7.94, *p* = 0.002). High staining patterns of ER-β1 conferred a marginally significant protective effect against developing metastases in the univariable model (HR 0.39, 95% CI 0.15–1.01, Mantel–Cox test *p* = 0.054, log-rank test *p* = 0.045, Figure 2), something that did not apply in the multivariable Cox models (Table 2).

With regard to OS, apart from statistically significant associations with T stage, N stage, and TNBC (compared to other molecular subtypes), we demonstrated a protective effect of high expression patterns of cytoplasmic E-cadherin (HR 0.37, 95% CI 0.18–0.77, *p* = 0.008) (Table 3).

**Figure 3:**
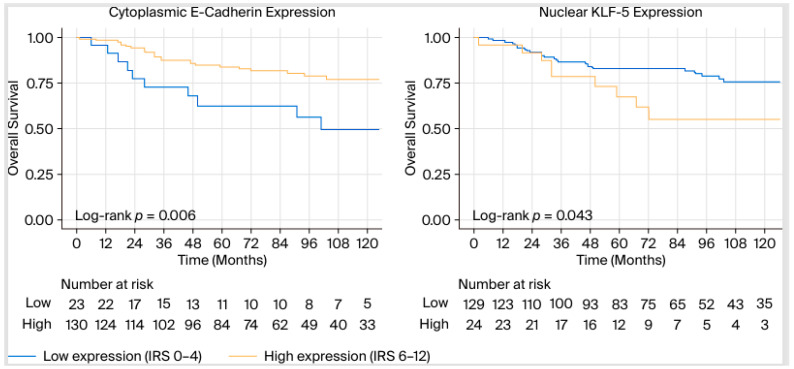
Expression of different molecules and their association with oncologic data. Kaplan–Meier curves and *p*-values corresponding to log-rank test between overall survival (OS) and cytoplasmic E-cadherin (left), nuclear KLF-5 expression (right).

Furthermore, high nuclear KLF-4 and KLF-5 expression were significantly associated with a worse OS compared with low expression patterns, *p* = 0.006 and *p* = 0.048, respectively. Figure 3 demonstrates the log-rank test between overall survival (OS) and cytoplasmic E-cadherin (left), nuclear KLF-5 expression (right).

**Figure 4:**
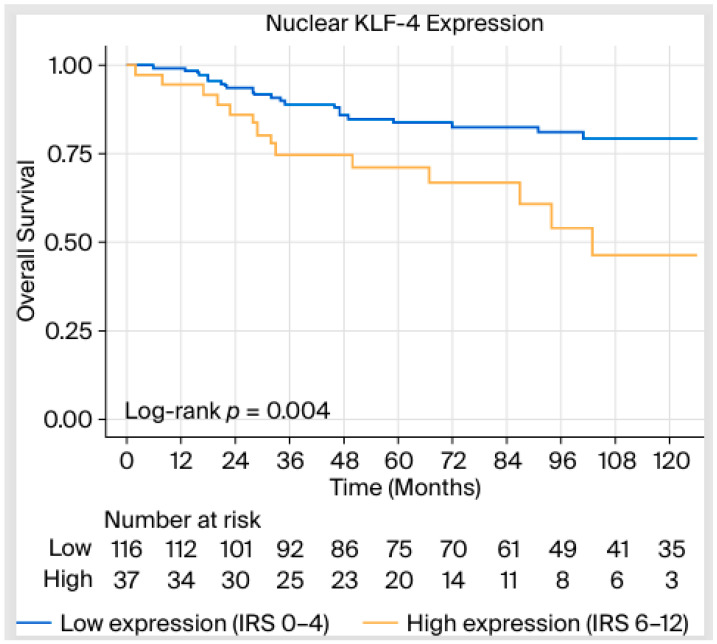
Kaplan–Meier curve and *p*-value corresponding to log-rank test between overall survival (OS) and nuclear KLF-4 expression.

In the multivariable analysis, the association of KLF-4 with worsened OS remained statistically significant (HR 4.09, 95% CI 1.93–8.67, *p* < 0.001) even after adjusting for T-, N stage, and molecular subtypes, along with the expression of β-catenin in the cytoplasm, E-cadherin in the cytoplasm, and KLF-5 in the nucleus. In summary, patients demonstrating a high expression of nuclear KLF-4 (IRS 6–12) had a 4.09-fold higher risk of death compared with those having a low expression (IRS 0–4) (Figure 4).

## 3. Discussion

In our retrospective cohort study, high nuclear expression of KLF-4 was significantly associated with reduced OS (univariable Cox regression *p* = 0.006), even after adjusting for established prognostic factors including tumor size, lymph node involvement, and molecular subtype (multivariable Cox regression *p* < 0.001). Furthermore, univariable Cox regression analyses further demonstrated a protective association with high E-cadherin expression (*p* = 0.008), whereas elevated levels of KLF-5 were linked to poorer OS (*p* = 0.048). These associations were not significant in the multivariable models. Additionally, ER-β1 expression was marginally associated with PFS and the development of metastases in the univariable (log-rank test *p* = 0.045) and multivariable analyses (multivariable Cox regression *p* = 0.059).

### 3.1. Association of Oncologic Outcomes and Molecular Expression of Different Proteins

In our study, ER-β1 expression was associated with a marginal improvement in PFS. The lack of statistical significance in PFS and OS may be partly due to limited power, underscoring the need for larger cohorts and improved stratification by BC subtypes. Additionally, variability in antibody selection for ER-β1 detection presents a potential confounding factor, complicating result interpretation. Nevertheless, our results are consistent with the established tumor-suppressive role of ER-β, which has been shown to antagonize ER-α signaling at least in vitro [29,30]. Mechanistically, this occurs through the formation of ER-α/ER-β heterodimers, which exhibit diminished transcriptional activity compared to ER-α homodimers, leading to decreased cellular proliferation and increased apoptosis, particularly in TNBC [29].

Preclinical data supports this mechanism. Mishra et al. demonstrated that treatment with fulvestrant, a selective estrogen receptor degrader (SERD), upregulated ERβ expression and concomitantly reduced proliferation in MDA-MB-231 TNBC cells [31]. In a related study, Zhao et al. employed LY500307, a synthetic ERβ agonist, to assess its impact on TNBC metastasis both in vitro and in vivo. Their findings revealed that pharmacological activation of ERβ not only reduced metastatic potential but also enhanced innate immune responses, thereby inhibiting pulmonary metastases. These results suggest that ERβ activation may represent a viable therapeutic strategy for patients with metastatic disease [32].

E-cadherin, a critical component for epithelial cell–cell adhesion, was significantly positively associated with improved OS (*p* = 0.008) in the univariable survival models. From a biological perspective, these findings align with the observations that loss of E-cadherin expression leads to EMT and loss of contact inhibition of proliferation. A variety of molecular pathways seem to be affected by altered E-cadherin signaling and may constitute potential therapeutic targets. These include Hippo, Wnt (including β-catenin), TGFβ, and NF-κB [33]. From a clinical perspective, the exact prognostic role of E-cadherin expression in BC survival outcomes is not clearly established [34,35,36,37,38]. For example, a large IHC analysis of more than 5000 BC cases demonstrated an association between E-cadherin low expression and lobular histology and tumors > 2 cm in size. No association between E-cadherin low expression and breast-specific survival outcomes could be observed [34]. Other smaller studies have provided heterogenous effect directions (i.e., positive/negative effects on survival) and associations with different parameters, such as lymph node involvement or response to chemotherapy [35,36,37,38].

KLFs are a wide group of 18 Cys2/zinc-finger proteins/transcription factors demonstrating a functional duality as both oncogenes and tumor suppressors depending on the cellular context [25,39]. In a functional genomic screen study, Rowland et al. demonstrated that KLF-4 regulates *TP53* activity depending on p21^CIP1^ status and cyclin D1 activity. More specifically, in cancer cell lines, KLF-4 allows for RAS^V12^-mediated transformation and escape of DNA-damage apoptosis [40]. Furthermore, knockdown mouse model studies demonstrated that KLF4 promotes BC oncogenesis through maintenance of cancer stem cells when overexpressed [41]. Similarly, KLF4 knockdown led to a reduction in cellular invasion, migration, and adhesion, actions mediated by the Notch signaling pathway [41]. Another possible involvement of KLF4 has been demonstrated by Moon and colleagues [42]. More specifically, KLF4 increased the expression of the platelet isoform of phosphofructokinase (PFKP), leading to high glycolytic metabolism [42]. Moreover, KLF-4 functions are modulated by interactions with other KLFs, such as KLF-5 [43].

KLF-5 also has a dual role in carcinogenesis. Its pro-proliferative activity is exerted by regulating protein-encoding genes [e.g., cell cycle genes cyclin A2 (*CCNA2*), *cyclin D1*, *FGF-BP1*, *Slug*, *tumor-necrosis factor-α* (*TNFα*)-*induced protein* (*TNFAIP2*)], and long-coding and microRNAs. As far as inhibitory effects are concerned, these include inhibition of proliferation and invasion attributed partially to post-translational modification by simulation and p53 activity, in a “switch” manner [25,44].

Preclinical evidence demonstrates that KLF-4 can act as a mediator of drug actions in BC tissues. Roberts et al. showed that KLF-4 represses epidermal growth factor receptor (EGFR) expression and downstream signaling in TNBC cells. Using transient overexpression and silencing of KLF-4 in MDA-MB-231, MDA-MB-468, and MCF10A cell lines, the authors showed that KLF-4 modulates cellular responses to the EGFR inhibitor erlotinib [45]. Another study identified KLF-4 and KLF-5 as cooperative regulators of drug resistance to lapatinib, a HER2 inhibitor, in HER2-enriched BC using genetically engineered C3(1) TAg and MMTV-Neu mice. These mechanisms were mediated in part by the regulation of the anti-apoptotic proteins myeloid cell leukemia 1 (MCL1) and B-cell lymphoma-extra-large (BCL-XL) [46].

Clinical evidence has examined the role of KLF-4 and KLF-5 expressions in survival outcomes in BC patients, leading to inconsistent results. Nagata et al. studied 208 BC patients and demonstrated that in TNBC, high expression of KLF-4 was associated with better disease-free survival (*p* = 0.0427) and OS (*p* = 0.0453) [47]. Subbalakshmi et al. demonstrated a statistically significant positive association between high KLF-4 expression and relapse-free survival (RFS) and OS [48]. Similarly, Zhu et al. confirmed the RFS benefit of high KLF-4 expression [49]. Takagi and colleagues examined the role of KLF-5 in BC-specific outcomes. More specifically, high IHC expression was associated with worse disease-free (*p* = 0.033) and BC-specific survival (*p* = 0.014). Mechanistically, KLF-5-dependent proliferative activity was androgen dependent showing a potential therapeutic target [50]. Apart from survival outcomes, surrogate markers such as pathological complete response (pCR) after chemotherapy have also been investigated [51]. More specifically, high KLF-4 expression has been associated with higher odds of non-pCR (OR 0.013, 95% CI 0.013–0.444, *p* = 0.004) [51].

Contrary to the above-mentioned literature, our study demonstrated a significant prognostic burden in patients with high expression (IRS 6–12) of KLF-4 and KLF-5. For the former, the association remained significant even after adjustment for classical factors affecting OS, including tumor size (T stage) or lymph node involvement (any). Patients with high expression of KLF-4 levels were at a 4.09-fold higher risk of death compared to those with low levels. Hence, the association described by the survival models constitutes a strong association requiring further investigation.

Apart from patient stratification, targeting KLFs for oncologic treatment has been studied in other tumor entities. More specifically, APTO-253, a c-Myc inhibitor and KLF-4 inducer, has been shown to lead to stable disease in 24% of patients with advanced solid tumors (i.e., colon cancer, other gastrointestinal malignancies, and lung cancer) in a Phase I clinical trial [52]. Regarding BC, APTO-253 treatment has been shown to induce apoptosis of TNBC cells lines via activation of *KLF-4* and *NOXA*, a proapoptotic factor belonging to the BCL-2 family [53]. Still, clinical trials of APTO-253 in BC patients are lacking.

### 3.2. Limitations

Despite the significance of the findings of this investigation, limitations should be considered. The use of tissue microarray (TMA) for assessing the expression levels of the studied molecules, while practical for high-throughput analysis, does not allow for a comprehensive evaluation of intratumoral heterogeneity or fully elucidate the functional roles of these markers in BC pathogenesis. Additionally, the study’s retrospective design limits the ability to establish causal relationships, and the associations observed should be interpreted with caution [54]. Further additional aspects that require addressing are the limited number of patients included, explained given the single-center character of the study, and potential variability in the performance of different antibodies and scoring methods for immunohistochemical methods, even though the expert pathologist assessment we followed mitigated the latter limitation. Prospective studies with experimental validation are warranted to confirm these findings and clarify the biological relevance of the investigated molecules.

### 3.3. Clinical Implications and Future Directions

Our single-center investigation provided preliminary evidence supporting the possible independent prognostic role of KLF4 in BC. These findings may contribute to a more nuanced stratification of patients beyond established molecular subtypes. Future studies should concentrate on multicentric prospective validation of KLF4 in large patient cohorts. Another interesting aspect constitutes the interactions of the studied molecules. In this regard, functional studies utilizing advanced molecular methodologies—such as proteomic and single-cell analyses—could provide deeper understanding of the mechanistic interplay governing BC pathogenesis. Furthermore, as explained in the section above, the potential of KLF4 as a therapeutic target is currently being evaluated in ongoing clinical studies at least in other oncologic entities. Further evidence, both preclinical and clinical, is required for such studies to be conducted in BC patients [52,53].

## 4. Materials and Methods

### 4.1. Study Design, Population, and Setting

This retrospective study included 153 female patients with histologically confirmed BC who underwent breast surgery between 2005 and 2020 at the Department of Gynecology, Obstetrics and Reproductive Medicine, Saarland University Medical Center, Homburg/Saar, Germany.

The inclusion criteria comprised the diagnosis of invasive ductal BC, the absence of distant metastases at the time of diagnosis, and no prior history of malignancies other than BC. Patient data and routine histopathological characteristics were comprehensively compiled using the SAP GUI C21 system (SAP Deutschland, Walldorf, Germany), the hospital’s software for patient management and data storage. For patients receiving neoadjuvant chemotherapy (NACT), a regression grading system according to Sinn was utilized to evaluate tumor response after NACT [55]. This system consists of five categories: score 0 indicates no evidence of therapeutic effect; score 1 is characterized by increased tumor fibrosis with inflammation and/or a distinct cytopathic effect; score 2 involves extensive tumor fibrosis with only small, localized areas of detectable tumor cells and multiple, minimal areas of residual tumor measuring 5 mm or less, often alongside a significant in situ component; score 3 reflects the absence of invasive residual tumor; and Score 4 denotes the complete absence of any residual tumor.

### 4.2. Construction of Tissue Microarrays (TMAs)

This part was carried out entirely at the Institute of General and Special Pathology, Saarland University Medical Center, Homburg/Saar, Germany. Paraffin-embedded tissue blocks from all included cases were obtained from the institute’s archives. All tumor blocks and archived sections were microscopically reevaluated. Representative tumor blocks were sectioned into 4 µm-thick slices and stained with hematoxylin and eosin (HE) to assess histological composition. Three representative tumor areas taken from the tumor center, each comprising at least 75% tumor cells, were identified. Following the marking of tumor regions on the original paraffin blocks, three core needle biopsies were obtained from each case. A total of eight tissue microarray (TMA) blocks were generated, with each block containing 60 cores derived from 20 cases. Subsequently, ten consecutive 4 µm-thick sections from each block were prepared and processed for HE-staining and immunohistochemistry (IHC) analysis.

### 4.3. Immunohistochemistry (IHC) Staining

Optimization of IHC staining was conducted in advance to determine the optimal antibody concentration, staining protocol, and experimental conditions. IHC staining of all analyzed proteins was conducted according to diagnostic standards and manufacturer instructions using the Dako REAL™ Detection System, Alkaline Phosphatase/RED, Rabbit/Mouse [Dako (Catalog No. K5005-DAKO)]. Primary antibodies, antigen retrieval, and blocking methods are listed in Table S4.

### 4.4. Quantification and Interpretation of IHC Staining

The assessment of staining results was performed by a board-certified pathologist (R.M.B.) under blind conditions to ensure precision and dependability. Representative images of stained sections were captured using a Zeiss Axioskop 40 microscope (Carl Zeiss, Oberkochen, Germany) equipped with a Carl Zeiss Axio Cam 208 Color camera for further analysis. The expression levels of all analyzed proteins were quantified according to the Remmele and Stegner immunoreactive scoring (IRS) system, based on staining intensity and the percentage of stained tumor cells [56].

Staining intensity was categorized as follows: negative (0), weak (1), moderate (2), and strong (3). The percentage of stained cells was classified as follows: 0 for no stained cells (0%), 1 for fewer than 10% stained cells, 2 for 10–50% stained cells, 3 for 51–80% stained cells, and 4 for more than 80% stained cells. The final IRS was determined by multiplying the staining intensity score by the percentage score. Based on these values, samples were divided into four groups: IRS 0–2, indicating negative staining; IRS 3–4, corresponding to weak staining; IRS 6–8, representing moderate staining; and IRS 9–12, signifying strong staining [56]. Furthermore, expression levels were dichotomized based on IRS values for further statistical analysis; cases with an IRS 0–4 were classified as low expression, while those with an IRS of 6–12 were considered high expression.

**Figure 5:**
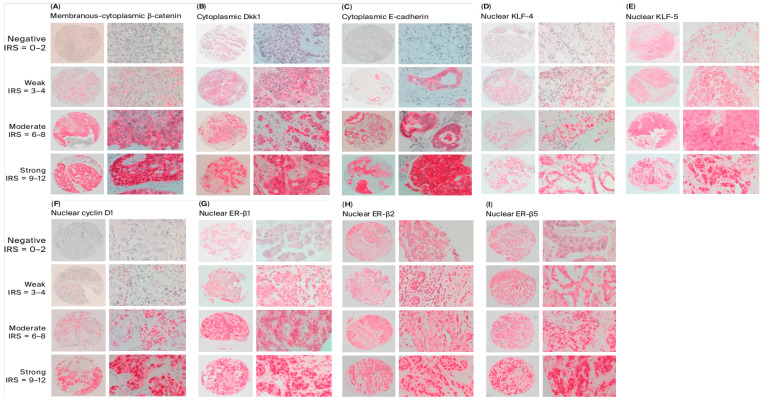
Immunohistochemistry (IHC) staining of tested proteins. Protein expression levels were categorized as negative, weak, moderate, or strong based on the immunoreactive score (IRS). (**A**) Cytoplasmic staining of β-catenin. (**B**) Cytoplasmic staining of Dkk1. (**C**) Cytoplasmic staining of E-Cadherin. (**D**) Nuclear staining of KFL-4. (**E**) Nuclear staining of KFL-5. (**F**) Nuclear staining of Cyclin D1: (**G**) Nuclear staining of ER-β1. (**H**) Nuclear staining of ER-β2. (**I**) Nuclear staining of ER-β5.

Cytoplasmic staining was evaluated for Dkk1, β-catenin, and E-cadherin, while nuclear staining was evaluated for ER-β1, ER-β2, ER-β5, cyclin D1, KLF-4, and KLF-5. Representative examples illustrating the categories of staining intensity are presented in Figure 5.

### 4.5. Statistical Analysis

Statistical analysis was conducted in SPSS (Version 29.0.2.0) and Jamovi (Version 2.3.28). For quantitative variables, values are presented as median (interquartile range). Qualitative variables are presented as absolute values (percentages, %).

The Kendall tau-b test was employed to assess correlations between the IRS expression levels of different molecules. Fisher’s exact test was used to assess differences in molecule expression (defined as low and high, as described above) in relation to different clinicopathologic characteristics.

The effects of different molecule expression levels on progression-free (PFS) and overall survival (OS) were evaluated by fitting Cox regression models and the Mantel–Cox test. Variables with *p*-values < 0.1 were then included in multivariable Cox regression models. Kaplan–Meier curves along with log-rank test results were constructed for significant associations between protein expression and survival outcomes.

Given that missing variables did not exceed 10% for each variable, no further measures were taken. Cases with missing data were not included in the analysis. Statistical significance was defined as *p* < 0.05, unless otherwise specified.

## 5. Conclusions

Our retrospective cohort TMA study provided evidence that high KLF-4 expression in IHC is associated with a worse OS in patients with BC. This effect remained statistically significant even after adjustment for T-stage, N- stage, and molecular subtypes along with E-cadherin, β-catenin, and KLF-5 expression. It provides preliminary evidence on the role KLF-4 in patient stratification in terms of diagnostics and requires further investigation in larger multicentric projects.

## Figures and Tables

**Table 1:** Demographic characteristics of the study population (*n* = 153).

Variable	Value	Missing
Age (years)	55.0 (47.0–64.0)	-
BMI (kg/m^2^)	25 (23–29.8)	5
Postmenopausal status	98 (64%)	-
Primary cancer (yes, recurrent/second *)	148 (96.7%), 5 (3.3%)	-
Molecular subtypes (luminal A, luminal B, Her2 positive, TNBC)	23 (15%), 36 (23.5%), 12 (7.9%), 82 (53.6%)	-
T-Stage (T1, T2, T3–4)	59 (38.8 %), 71 (46.7 %), 22 (14.5 %)	1
N-Stage (N0, N1–3)	78 (52%), 72 (48%)	3
G-Stage (G1–2, G3)	67 (44.7%), 83 (55.3%)	3
Ki67-Index (≤14%, >14%)	27 (18.9%), 116 (81.1%)	10
NACT	85 (55.6%)	-
Operation type (breast conserving surgery, mastectomy)	114 (74.5%), 39 (25.5%)	-

*n*: Number of patients. * Three cases had a second breast cancer diagnosis (first diagnosed in 1992, 1997, and 2005), while two cases experienced a recurrence (first case occurred in 2003 and 2004). BMI: body mass index, NACT: neoadjuvant chemotherapy, TNBC: triple negative breast cancer. Data are represented as numbers and percentages.

**Table 2:** Univariable and multivariable Cox regression models for progression-free survival (PFS).

PFS	Univariable Cox Regression		Multivariable Cox Regression	
	HR (95% CI)	*p*	HR (95% CI)	*p*
Menopausal status				
Pre/perimenopausal	1	-	-	-
Postmenopausal	1.04 (0.53–2.06)	0.906	-	-
T-stage				
T1	1	-	1	-
T2–4	2.30 (1.08–4.90)	0.031	1.86 (0.84–4.09)	0.125
N-Stage				
N0	1	-	1	-
N1–3	4.27 (1.97–9.28)	<0.001	5.17 (2.33–11.48)	<0.001
Molecular subtypes				
Other types (all)	1	-	1	-
TNBC	3.60 (1.63–7.94)	0.002	4.79 (2.13–10.78)	<0.001
Cytoplasmic Dkk1 expression				
Low expression (IRS 0–4)	1	-	-	-
High expression (IRS 6–12)	0.60 (0.31–1.17)	0.136	-	-
Cytoplasmic β-catenin expression				
Low expression (IRS 0–4)	1	-	-	-
High expression (IRS 6–12)	0.99 (0.43–2.25)	0.972	-	-
Cytoplasmic E cadherin expression				
Low expression (IRS 0–4)	1	-	-	-
High expression (IRS 6–12)	0.73 (0.31–1.68)	0.454	-	-
Nuclear KLF-4 expression				
Low expression (IRS 0–4)	1	-	-	-
High expression (IRS 6–12)	0.89 (0.39–2.03)	0.775	-	-
Nuclear KLF-5 expression				
Low expression (IRS 0–4)	1	-	-	-
High expression (IRS 6–12)	1.64 (0.71–3.78)	0.246	-	-
Nuclear Cyclin D1 expression				
Low expression (IRS 0–4)	1	-	-	-
High expression (IRS 6–12)	0.55 (0.19–1.56)	0.263	-	-
Nuclear ER-β1 expression				
Low expression (IRS 0–4)	1	-	1	-
High expression (IRS 6–12)	0.39 (0.15–1.01)	0.054	0.39 (0.15–1.04)	0.059
Nuclear ER-β2 expression				
Low expression (IRS 0–4)	1	-	-	-
High expression (IRS 6–12)	0.54 (0.24–1.25)	0.152	-	-
Nuclear ER-β5 expression				
Low expression (IRS 0–4)	1	-	-	-
High expression (IRS 6–12)	1.49 (0.52–4.29)	0.458	-	-

CI: confidence interval, HR: hazard ratio, IRS: immunoreactive score, PFS: progression-free survival, TNBC: triple negative breast cancer.

**Table 3:** Univariable and multivariable Cox regression models for overall survival (OS).

OS	Univariable Cox Regression		Multivariable Cox Regression	
	HR (95% CI)	*p*	HR (95% CI)	*p*
Menopausal status				
Pre/perimenopausal	1	-	-	-
Postmenopausal	1.77 (0.82–3.80)	0.143	-	-
T-stage				
T1	1	-	1	-
T2–4	3.56 (1.47–8.61)	0.005	4.61 (1.61–13.21)	0.004
N-Stage				
N0	1	-	1	-
N1–3	3.64 (1.64–8.06)	0.001	4.38 (1.85–10.41)	0.001
Molecular subtypes				
Other types (all)	1	-	1	-
TNBC	4.93 (2.04–11.92)	<0.001	7.17 (2.51–20.50)	<0.001
Cytoplasmic Dkk1 expression				
Low expression (IRS 0–4)	1	-	-	-
High expression (IRS 6–12)	0.92 (0.47–1.80)	0.806	-	-
Cytoplasmic β-catenin expression				
Low expression (IRS 0–4)	1	-	1	-
High expression (IRS 6–12)	0.54 (0.26–1.12)	0.099	0.60 (0.22–1.61)	0.309
Cytoplasmic E cadherin expression				
Low expression (IRS 0–4)	1	-	1	-
High expression (IRS 6–12)	0.37 (0.18–0.77)	0.008	0.40 (0.15–1.08)	0.069
Nuclear KLF-4 expression				
Low expression (IRS 0–4)	1	-	1	-
High expression (IRS 6–12)	2.63 (1.32–5.22)	0.006	4.09 (1.93–8.67)	<0.001
Nuclear KLF-5 expression				
Low expression (IRS 0–4)	1	-	1	-
High expression (IRS 6–12)	2.16 (1.01–4.65)	0.048	1.20 (0.51–2.85)	0.673
Nuclear Cyclin D1 expression				
Low expression (IRS 0–4)	1	-	-	-
High expression (IRS 6–12)	0.43 (0.13–1.42)	0.166	-	-
Nuclear ER-β1 expression				
Low expression (IRS 0–4)	1	-	-	-
High expression (IRS 6–12)	0.45 (0.16–1.29)	0.139	-	-
Nuclear ER-β2 expression				
Low expression (IRS 0–4)	1	-	-	-
High expression (IRS 6–12)	0.71 (0.27–1.84)	0.479	-	-
Nuclear ER-β5 expression				
Low expression (IRS 0–4)	1	-	-	-
High expression (IRS 6–12)	0.94 (0.36–2.45)	0.906	-	-

CI: confidence interval, HR: hazard ratio, IRS: immunoreactive score, OS: overall survival, TNBC: triple negative breast cancer.

## Data Availability

The data collected during this study are available from the corresponding author on reasonable request.

## Supplementary Materials

The following supporting information can be downloaded at https://www.mdpi.com/article/10.3390/ijms27062576/s1.

## Abbreviations

The following abbreviations are used in this manuscript:

BC	breast cancer
CDK4/6	cyclin-dependent kinases 4 and 6
Dkk1	Dickkopf-1
EMT	epithelial-mesenchymal transition
HE	hematoxylin and eosin
IHC	immunohistochemistry
IRS	immunoreactive scoring
KLF	Krüppel-like factors
NACT	neoadjuvant chemotherapy
OS	overall survival
PARPi	poly (ADP-ribose) polymerase inhibitors
PD-1	programmed death-1
PFS	progression-free survival
SERD	selective estrogen receptor degrader
TMAs	tissue microarrays
TNBC	triple negative breast cancer
