# Supplementary material for: Multi-Protein Profiling Reveals High Nuclear KFL-4 Expression as a Predictor of Poor Overall Survival in Breast Cancer: A Retrospective Cohort Study
Source: Int J Mol Sci. 2026 Mar 11;27(6):2576. doi: 10.3390/ijms27062576 (PMC13026467; doi:10.3390/ijms27062576)
Supplement: Supplementary file 1 [file ijms-27-02576-s001.zip › ijms-4113589-supplementary.pdf]

Supplementary Material

Journal: International Journal of Molecular Sciences

Manuscript: Multi-protein profiling reveals high nuclear KFL-4 expression as a predictor of poor overall survival in breast cancer: a retro-spective cohort study

Authors
Mariz Kasoha[1], Bashar Haj Hamoud[1], Rainer M. Bohle[2], Barbara Linxweiler[1], Raphaela Bosch[1], Gilbert Georg Klamminger[3], Gilda Schmidt[1], Erich-Franz Solomayer[1], Meletios P. Nigdelis[1]

Affiliations:
[1] Department of Gynecology, Obstetrics and Reproductive Medicine, Saarland University Medical Center, 66421 Homburg, Germany
[2] Institute of General and Surgical Pathology, University Medical School of Saarland, 66421 Homburg, Germany
[3] Department of Obstetrics and Gynecology, University Medical Center of the Johannes Gutenberg University Mainz, 55131 Mainz, Germany

Table S1. Expression levels of different molecules based on IRS staining patters according to Remmele and Stegner [1].

	Negative staining IRS=0-2	Weak staining IRS=3-4	Moderate staining IRS=6-8	Strong staining IRS=9-12
Dkk1 (Cyt)	12 (7.8%)	62 (40.5%)	68 (44.4%)	11 (7.2%)
β-Catenin (Cyt)	11(7.2%)	20 (13.1%)	61 (39.9%)	61 (39.9%)
E-cadherin (Cyt)	5 (3.3%)	18 (11.8%)	75 (49.0%)	55 (35.9%)
KLF-4 (N)	72 (47.1%)	44 (28.8%)	35 (22.9%)	2 (1.3%)
KLF-5 (N)	99 (64.7%)	30 (19.6%)	22 (14.4%)	2 (1.3%)
Cyclin D (N)	98 (64.1%)	28 (18.3%)	21 (13.7%)	6 (3.9%)
PR (N)	116 (75.8%)	7 (4.6%)	17 (11.1%)	13 (8.5%)
ER-α (N)	94 (61.4%)	7 (4.6%)	16 (10.5%)	36 (23.5%)
ER-β1 (N)	7 (4.6%)	4 (2.6%)	46 (30.1%)	96 (62.7%)
ER-β2 (N)	5 (3.3%)	12 (7.8%)	47 (30.7%)	89 (58.2%)
ER-β5 (N)	3 (2.0%)	17 (11.1%)	88 (57.5%)	45 (29.4%)

Cyt: cytoplasmic, ER: estrogen receptor, IRS: immunoreactive score according to Remmele and Stegner, KLF: Krüppel-like factor, N: nuclear, PR: progesterone receptor. Data are represented as numbers and percentages.

References

1. Remmele W, Stegner HE. [Recommendation for uniform definition of an immunoreactive score (IRS) for immunohistochemical estrogen receptor detection (ER-ICA) in breast cancer tissue]. Pathologe. 1987;8.

Table S2. Correlation analysis between expression levels (IRS scores) of different molecules.

		KLF-5 (N)	Dkk1 (Cyt)	β-Catenin (Cyt)	E-cadherin (Cyt)	Cyclin D1 (N)	PR (N)	ER-α (N)	ER-β1 (N)	ER-β2 (N)	ER-β5 (N)
KLF-4	CC	0.127	0.085	0.046	-0.014	0.127	0.122	0.047	0.179	0.236	0.125
(N)	*p*	0.052	0.181	0.465	0.824	0.050	0.065	0.480	0.006	<0.001	0.055
KLF-5	CC	---	-0.129	0.112	0.017	-0.018	-0.230	-0.250	0.111	0.074	0.166
(N)	*p*		0.051	0.087	0.800	0.793	<0.001	<0.001	0.101	0.270	0.014
Dkk1	CC		---	0.006	0.117	0.232	0.200	0.259	0.149	0.223	0.186
(Cyt)	*p*			0.925	0.072	<0.001	0.003	<0.001	0.024	0.001	0.005
β-Catenin	CC			---	0.439	0.057	0.029	-0.025	0.201	0.035	0.140*
(Cyt)	*p*				<0.001	0.383	0.663	0.708	0.002	0.591	0.032
E-cadherin	CC				---	0.161	0.110	0.135	0.145	0.071	0.162
(Cyt)	*p*					0.015	0.104	0.046	0.029	0.276	0.014
Cyclin D1	CC					---	0.301	0.418	0.254	0.301	0.169
(N)	*p*						<0.001	<0.001	<0.001	<0.001	0.011
PR	CC						---	0.684	0.197	0.201	-0.089
(N)	*p*							<0.001	0.004	0.003	0.194
ER-α	CC							---	0.271	0.309	-0.061
(N)	*p*								<0.001	<0.001	0.369
ER-β1	CC								---	0.404	0.344
(N)	*p*									<0.001	<0.001
ER-β2	CC									---	0.283
(N)	*p*										<0.001
ER-β5	CC										---
(N)	*p*										

p-values were calculated using Kendall-Tau-b test. Data is presented as correlation coefficient (CC) and *p* values. Cyt: cytoplasmic, ER: estrogen receptor, KLF: Krüppel-like factor, N: nuclear, PR: progesterone receptor.

Table S3. Associations between clinicopathological parameters and different protein expressions levels. Low expression was defined as IRS scores 0-4, while high expression was defined as IRS scores 6-12.

		**Dkk1 Cytoplasmic**			**β-Catenin Cytoplasmic**			**E-Cadherin Cytoplasmic**		
		Low expression	High expression	*p*	Low expression	High expression	*p*	Low expression	High expression	*p*
T-Stage	T1	27 (46%)	32 (54%)	0.619	13 (22%)	46 (78%)	0.676	8 (14%)	51 (86%)	0.817
	T2-3-4	47 (51%)	46 (49%)		17 (18%)	76 (82%)		15 (16%)	78 (84%)	
N-Stage	N0	40 (51%)	38 (49%)	0.628	16 (21%)	62 (79%)	0.837	13 (17%)	65 (83%)	0.658
	N1-2-3	34 (47%)	38 (53%)		13 (18%)	59 (82%)		10 (14%)	62 (86%)	
G-Stage	G1-2	30 (45%)	37 (55%)	0.415	10 (15%)	57 (85%)	0.218	6 (9%)	61 (91%)	0.068
	G3	43 (52%)	40 (48%)		20 (24%)	63 (76%)		17 (21%)	66 (79%)	
Ki67-Index (%)	≤14	11 (41%)	16 (59%)	0.396	6 (22%)	21 (78%)	0.788	4 (15%)	23 (85%)	1.000
	>14	59 (51%)	57 (49%)		22 (19%)	94 (81%)		16 (14%)	100 (86%)	
Molecular subtypes	Luminal A	9 (39%)	14 (61%)	0.002	5 (22%)	18 (78%)	1.000	3 (13%)	20 (87%)	0.011
	Luminal B	10 (28%)	26 (72%)		7 (19%)	29 (81%)		1 (3%)	35 (97%)	
	Her2 positive	4 (33%)	8 (67%)		2 (17%)	10 (83%)		0 (0%)	12 (100%)	
	TNBC	51 (62%)	31 (38%)		17 (21%)	65 (79%)		19 (23%)	63 (77%)	
	Other types (all)	23 (32%)	48 (68%)	<0.001	14 (20%)	57 (80%)	1.000	4 (6%)	67 (94%)	0.003
	TNBC	51 (62%)	31 (38%)		17 (21%)	65 (79%)		19 (23%)	63 (77%)	
PFS status	Did not develop M	52 (44%)	65 (56%)	0.089	24 (21%)	93 (79%)	1.000	16 (14%)	101 (86%)	0.427
	Developed M	22 (61%)	14 (39%)		7 (19%)	29 (81%)		7 (19%)	29 (81%)	
OS status	Alive	57 (48%)	62 (52%)	0.848	21 (18%)	98 (82%)	0.150	13 (11%)	106 (89%)	0.013
	Died	17 (50%)	17 (50%)		10 (29%)	24 (71%)		10 (29%)	24 (71%)	

M: metastasis, OS: overall survival, PFS: progression-free survival, TNBC: triple negative breast cancer. The p-values correspond to Fisher's exact test. Data presented as number of cases and percentage.

Table S3. (continued)

		KLF-4 Nuclear			KLF-5 Nuclear			Cyclin D1 Nuclear		
		Low expression	High expression	*p*	Low expression	High expression	*p*	Low expression	High expression	*p*
T-Stage	T 1	41 (70%)	18 (30%)	0.123	46 (78%)	13 (22%)	0.112	49 (83%)	10 (17%)	1.000
	T 2-3-4	75 (81%)	18 (19%)		82 (88%)	11 (12%)		77 (83%)	16 (17%)	
N-Stage	N0	59 (76%)	19 (24%)	0.848	63 (81%)	15 (19%)	0.276	65 (83%)	13 (17%)	1.000
	N 1-2-3	56 (78%)	16 (22%)		63 (88%)	9 (12%)		60 (83%)	12 (17%)	
G-Stage	G 1-2	52 (78%)	15 (22%)	0.705	63 (94%)	4 (6%)	0.003	51 (76%)	16 (24%)	0.082
	G3	62 (75%)	21 (25%)		63 (76%)	20 (24%)		73 (88%)	10 (12%)	
Ki67-Index (%)	≤14	18 (67%)	9 (33%)	0.214	27 (100%)	0 (0%)	0.014	21 (78%)	6 (22%)	0.594
	>14	91 (78%)	25 (22%)		95 (82%)	21 (18%)		95 (82%)	21 (18%)	
Molecular subtypes	Luminal A	15 (65%)	8 (35%)	0.219	23 (100%)	0 (0%)	<0.001	17 (74%)	6 (26%)	<0.001
	Luminal B	29 (81%)	7 (19%)		34 (94%)	2 (6%)		18 (50%)	18 (50%)	
	Her2 positive	7 (58%)	5 (42%)		12 (100%)	0 (0%)		10 (83%)	2 (17%)	
	TNBC	65 (79%)	17 (21%)		60 (73%)	22 (27%)		81 (99%)	1 (1%)	
	Other types (all)	51 (72%)	20 (28%)	0.345	69 (97%)	2 (3%)	<0.001	45 (63%)	26 (37%)	<0.001
	TNBC	65 (79%)	17 (21%)		60 (73%)	22 (27%)		81 (99%)	1 (1%)	
PFS status	Did not develop M	87 (74%)	30 (26%)	0.511	100 (86%)	17 (14%)	0.446	94 (80%)	23 (20%)	0.320
	Developed M	29 (81%)	7 (19%)		29 (81%)	7 (19%)		32 (89%)	4 (11%)	
OS status	Alive	96 (81%)	23 (19%)	0.012	104 (87%)	15 (13%)	0.062	95 (80%)	24 (20%)	0.200
	Died	20 (59%)	14 (41%)		25 (74%)	9 (26%)		31 (91%)	3 (9%)	

KLF: Krüppel-like factor. M: metastasis, OS: overall survival, PFS: progression-free survival, TNBC: triple negative breast cancer. The p-values correspond to Fisher's exact test. Data presented as number of cases and percentage.

Table S3. (continued)

		ER-β1 Nuclear			ER-β2 Nuclear			ER-β5 Nuclear		
		Low expression	High expression	*p*	Low expression	High expression	*p*	Low expression	High expression	*p*
T-Stage	T1	4 (7%)	55 (93%)	1.000	6 (10%)	53 (90%)	0.799	7 (12%)	52 (88%)	0.808
	T2-3-4	7 (8%)	86 (92%)		11 (12%)	82 (88%)		13 (14%)	80 (86%)	
N-Stage	N0	6 (8%)	72 (92%)	1.000	8 (10%)	70 (90%)	0.798	11 (14%)	67 (86%)	0.814
	N1-2-3	5 (7%)	67 (93%)		9 (13%)	63 (87%)		9 (13%)	63 (87%)	
G-Stage	G1-2	3 (5%)	64 (95%)	0.347	6 (9%)	61 (91%)	0.449	8 (12%)	59 (88%)	0.810
	G3	8 (10%)	75 (90%)		11 (13%)	72 (87%)		12 (15%)	71 (85%)	
Ki67-Index (%)	≤14	0 (0%)	27 (100%)	0.125	0 (0%)	27 (100%)	0.042	7 (26%)	20 (74%)	0.053
	>14	11 (10%)	105 (90%)		16 (14%)	100 (86%)		12 (10%)	104 (90%)	
Molecular subtypes	Luminal A	0 (0%)	23 (100%)	0.127	0 (0%)	23 (100%)	0.025	5 (22%)	18 (78%)	0.295
	Luminal B	1 (3%)	35 (97%)		2 (6%)	34 (94%)		2 (6%)	34 (94%)	
	Her2 positive	0 (0%)	12 (100%)		0 (0%)	12 (100%)		1 (8%)	11 (92%)	
	TNBC	10 (12%)	72 (88%)		15 (18%)	67 (82%)		12 (15%)	70 (85%)	
	Other types (all)	1 (1%)	70 (99%)	0.011	2 (3%)	69 (97%)	0.003	8 (11%)	63 (89%)	0.634
	TNBC	10 (12%)	72 (88%)		15 (18%)	67 (82%)		12 (15%)	70 (85%)	
PFS status	Did not develop M	6 (5%)	111 (95%)	0.131	10 (9%)	107 (91%)	0.124	16 (14%)	101 (86%)	0.785
	Developed M	5 (14%)	31 (86%)		7 (19%)	29 (81%)		4 (11%)	32 (89%)	
OS status	Alive	7 (6%)	112 (94%)	0.263	12 (10%)	107 (90%)	0.536	15 (13%)	104 (87%)	0.775
	Died	4 (12%)	30 (88%)		5 (15%)	29 (85%)		5 (15%)	29 (85%)	

ER: estrogen receptor. M: metastasis, OS: overall survival, PFS: progression-free survival, TNBC: triple negative breast cancer. The p-values correspond to Fisher's exact test. Data presented as number of cases and percentage.

Table S4. Immunohistochemical staining primary antibodies and conditions.

Primary antibodies				Antigen retrieval		Blocking	
Antibody	Supplier	Catalog No.	Titre	PH	Time (Min)	Buffer	Time (Min)
β-Catenin	abcam	ab32572	1/600	6	25	3% PBS	50
Dkk1	abcam	ab109416	1/800	9	5	5%PBS+Tween	50
KLF-4	abcam	ab215036	1/1000	9	13	5%PBS+Tween	60
KLF-5	abcam	ab137676	1/500	9	13	5%PBS+Tween	60
Cyclin D1	Cell Signalling Technology	#55506	1/800	6	25	3% PBS	50
E-Cadherin	Cell Signalling Technology	#3195	1/400	6	25	3% PBS	50
Estrogen receptor beta 1	BIO-RAD	MCA 1974GA	1/150	6	25	3% PBS	50
Estrogen receptor beta 2	BIO-RAD	MCA 2279GT	1/500	6	25	3% PBS	50
Estrogen receptor beta 5	BIO-RAD	MCA 4676T	1/75	6	25	3% PBS	50

PBS: Phosphate-buffered saline
